# Diagnostic value of potassium level in a spot urine sample as an index of 24-hour urinary potassium excretion in unselected patients hospitalized in a hypertension unit

**DOI:** 10.1371/journal.pone.0180117

**Published:** 2017-06-29

**Authors:** Piotr Jędrusik, Bartosz Symonides, Ewa Wojciechowska, Adam Gryglas, Zbigniew Gaciong

**Affiliations:** 1 Department of Internal Medicine, Hypertension and Vascular Diseases, Medical University of Warsaw, Warsaw, Poland; 2 Student Society at the Department of Internal Medicine, Hypertension and Vascular Diseases, Medical University of Warsaw, Warsaw, Poland; The University of Tokyo, JAPAN

## Abstract

**Background:**

Primary hyperaldosteronism may be associated with elevated 24-hour urinary potassium excretion. We evaluated the diagnostic value of spot urine (SU) potassium as an index of 24-hour urinary potassium excretion.

**Methods:**

We measured SU and 24-hour urinary collection potassium and creatinine in 382 patients. Correlations between SU and 24-hour collections were assessed for potassium levels and potassium/creatinine ratios. We used the PAHO formula to estimate 24-hour urinary potassium excretion based on SU potassium level. The agreement between estimated and measured 24-hour urinary potassium excretion was evaluated using the Bland-Altman method. To evaluate diagnostic performance of SU potassium, we calculated areas under the curve (AUC) for SU potassium/creatinine ratio and 24-hour urinary potassium excretion estimated using the PAHO formula.

**Results:**

Strongest correlation between SU and 24-hour collection was found for potassium/creatinine ratio (r = 0.69, P<0.001). The PAHO formula underestimated 24-hour urinary potassium excretion by mean 8.3±18 mmol/d (95% limits of agreement -28 to +44 mmol/d). Diagnostic performance of SU potassium/creatinine ratio was borderline good only if 24-hour urinary potassium excretion was largely elevated (AUC 0.802 for 120 mmol K^+^/24 h) but poor with lower values (AUC 0.696 for 100 mmol K^+^/24 h, 0.636 for 80 mmol K^+^/24 h, 0.675 for 40 mmol K^+^/24 h). Diagnostic performance of 24-hour urinary potassium excretion estimated by the PAHO formula was excellent with values above 120 mmol/d and good with lower values (AUC 0.941 for 120 mmol K^+^/24 h, 0.819 for 100 mmol K^+^/24 h, 0.823 for 80 mmol K^+^/24 h, 0.836 for 40 mmol K^+^/24 h).

**Conclusions:**

Spot urine potassium/creatinine ratio might be a marker of increased 24-hour urinary potassium excretion and a potentially useful screening test when reliable 24-hour urine collection is not available. The PAHO formula allowed estimation of the 24-hour urinary potassium excretion based on SU measurements with reasonable clinical accuracy.

## Introduction

Hypokalemia due to an increased urinary potassium excretion is a feature of primary hyperaldosteronism (PHA), a disorder that is increasingly recognized as one of the most prevalent forms of secondary hypertension [1]. Although increased urinary potassium loss and resultant hypokalemia are not seen in as many as 60% of patients with PHA [2], they are more common in patients with an adrenal adenoma which is potentially curable by adrenalectomy. In a large Italian study, hypokalemia was found in 50% of patients with an aldosterone-producing adenoma compared to only 17% of patients with bilateral hyperplasia [3]. In addition, some of the presenting symptoms in patients with PHA are related to hypokalemia, such as fatigue, muscle weakness, and paresthesiae. However, hypokalemia may result from alternative causes such as potassium losses caused by diuretics or gastrointestinal disorders (e.g., diarrhea or laxative abuse), and these conditions may also be common in hypertensive patients. Thus, measurement of urine potassium may help establish the pathophysiologic mechanism behind hypokalemia and aid in formulating the differential diagnosis.

An established gold standard diagnostic tool to evaluate urinary potassium excretion is 24-hour urine collection. However, 24-hour urine collection is cumbersome and labor intensive, particularly in outpatient settings, and in some patients may be even impossible. It is often unreliable due to difficulties with obtaining a complete 24-hour collection, resulting in both under- and overcollection of samples, and no methods are available to accurately identify incomplete samples [4]. In large population studies, usefulness of 24-hour urine collection has been also limited by low response rates [4]. In contrast, single (spot) urine samples are easily collected and stored without a potential for under- or overcollection. Spot urine samples are widely used to detect and monitor proteinuria [5] and have been evaluated for the potential of assessing urinary sodium excretion, mostly for the purpose of estimating population salt intake [4,6].

Several formulae have been developed to estimate 24-hour urinary excretion of sodium and potassium based on the measured sodium or potassium level in a spot urine sample. Two such formulas were developed using regression analysis and validated in Japanese populations [7,8], and a simpler, more general formula has been proposed by the Pan American Health Organization (PAHO) and the World Health Organization to estimate 24-hour urinary excretion of sodium, potassium and iodine [9]. However, most published reports are from population studies that were performed in healthy subjects and none of these formulas were specifically evaluated in terms of their diagnostic value for estimating 24-hour urinary potassium excretion in hypertensive patients.

The aim of our study was to evaluate the diagnostic value of potassium level measurement in a spot urine sample as an index of 24-hour urinary potassium excretion in unselected patients hospitalized in a hypertensive unit, either expressed as the potassium to creatinine ratio or used to estimate 24-hour urinary potassium excretion using an available formula.

## Material and methods

We studied 382 patients hospitalized in our department who were ordered a routine 24-hour urine collection for various reasons, i.e., to estimate glomerular filtration rate (GFR), measure urinary protein excretion, estimate sodium intake, or measure 24-hour urinary potassium excretion as a part of the diagnostic work-up for secondary hypertension.

A spot urine sample was taken in the morning, in the fasting conditions and before administration of medications, and sent to the hospital laboratory for routine urinalysis along with determination of sodium, potassium, and creatinine level, followed by 24-hour urinary collection until the next morning. The volume of the 24-hour urinary collection was measured, and analytes determined in the 24-hour urinary collection included sodium, potassium, and creatinine level.

All patients provided morning urine samples and underwent 24-hour urinary collection during their inpatient stay in our hypertension unit, usually started within 24 hours after admission. The patient were given standard verbal instructions by the nursing personnel on how to collect a complete 24-hour urinary collection and given a 2000 mL container that was kept by the patient until next morning. If additional containers were needed due to a larger urine volume, they were provided by the nursing personnel. Apart from prior verbal instruction, the completeness of urine collection was not directly monitored by the hospital staff during the collection period.

Exclusion criteria were: oliguria (24-hour urine collection volume < 400 mL), polyuria (24-hour urine collection volume > 3000 mL), and unreliable 24-hour urine collection as indicated by 24-hour urinary creatinine excretion of < 0.6 g/day (based on average 24-hour urinary creatinine excretion of 14–26 mg/kg/day, or 0.6–2.0 g/day) [10].

Urinary sodium, potassium, and creatinine levels were measured using standard laboratory methods (Cobas Integra analyzers, Roche). GFR was estimated using the Modification of Diet in Renal Disease (MDRD) formula. All patients underwent clinical evaluation that included medical history taking, detailed physical examination, office blood pressure measurements, and 24-hour ambulatory blood pressure monitoring. In all patients, various diagnostic tests were also performed as deemed clinically required for the evaluation of hypertension, including plasma aldosterone-renin ratio, blood and 24-hour urine aldosterone levels, 24-hour catecholamine and their metabolite excretion, and imaging studies of the kidneys, adrenals and renal arteries.

Patient informed consent and study approval by the Ethics Committee was not required, as all procedures (obtaining a spot urine sample and a 24-hour urinary collection) were a part of the routine diagnostic work-up that was indicated on clinical grounds and samples were anonymized. No need for ethical approval was formally confirmed by the local Ethics Committee at our institution. Data were collected in 2006–2012.

Correlations between laboratory parameters in the spot urine sample and 24-hour urine collection were assessed using Pearson correlation coefficient. As the amount of creatinine excreted daily is fairly constant across the population regardless of potassium excretion, as it mainly depends on lean body mass, we hypothesized that the potassium to creatinine ratio in a spot urine sample might represent a useful measure of 24-hour urinary potassium excretion. For estimating 24-hour urinary potassium excretion based on potassium level in the spot urine sample, we used the following formula endorsed by PAHO [9,11]:

Estimated 24-hour potassium excretion (mmol/24 h) = (measured spot urine potassium level / measured spot urine creatinine level) x measured 24-hour creatinine excretion

The agreement between estimated and measured 24-hour urinary potassium excretion was evaluated using the Pearson correlation coefficient and the Bland-Altman approach [12,13]. In the latter approach, the mean bias is the mean difference between the measured and estimated 24-hour urinary potassium excretion in the study population. To obtain the mean bias, the difference between the measured and predicted value was calculated for each participant, and then a mean of these individual differences was calculated. A Bland-Altman plot was used to evaluate the individual differences between measured and predicted urinary potassium excretion. The X axis of the Bland-Altman plot shows the individual means of the measured and estimated 24-hour urinary potassium excretion, and the Y axis shows the differences between the two methods, obtained by subtracting the estimated results from the measured 24-hour urine collection results. The 95% limit of agreement is estimated as the mean bias ±1.96 standard deviation of the mean bias.

To evaluate diagnostic performance of potassium level in a spot urine sample, we calculated areas under the curve (AUC) for receiver-operating characteristic (ROC) curves created for spot urine potassium level and potassium/creatinine ratio, and 24-hour urinary potassium excretion estimated using the PAHO formula. ROC curves were plotted for the following cut-off values of the measured 24-hour urinary potassium excretion: 40, 60, 80, 100, and 120 mmol/d. All statistical analyses were performed using the R software. P<0.05 was considered statistically significant.

## Results

The study group included 152 men and 230 women. The mean age was 55±16 years (range 16–94 years), and the mean body mass index was 28.9±5.4 kg/m^2^. Hypertension was present in 92% of patients. Study group characteristics are shown in Table 1.

**Table 1:** Study group characteristics.

Variable	Study population (N = 382)
Gender (male/female)	152/230
Mean age (range) [years]	55±16 (range 16–94)
Mean body mass index [kg/m^2^]	28.9±5.4
Hypertension	92%
Cardiovascular disease	29%
Dysglycemia (diabetes, impaired glucose tolerance or impaired fasting glucose)	23%
Mean 24-hour urinary potassium excretion [mmol/d]	54±24
Mean 24-hour urinary creatinine excretion [g/d]	1.23±0.49
Mean serum creatinine level [mg/dL]	0.95±0.48
Estimated GFR < 60 mL/min/1.73 m^2^	17%
Mean clinic blood pressure [mm Hg]	152±29/91±15
Mean ambulatory daytime blood pressure [mm Hg]	132±18/78±12
Mean ambulatory nighttime blood pressure [mm Hg]	121±21/62±12
Any hypertensive medication	92%
Loop diuretic or thiazide	45%
Spironolactone	4.5%
Hydrochlorothiazide/amiloride	1.8%
ACE inhibitor/angiotensin receptor blocker	60%
Potassium supplementation	45%

**Fig 1:**
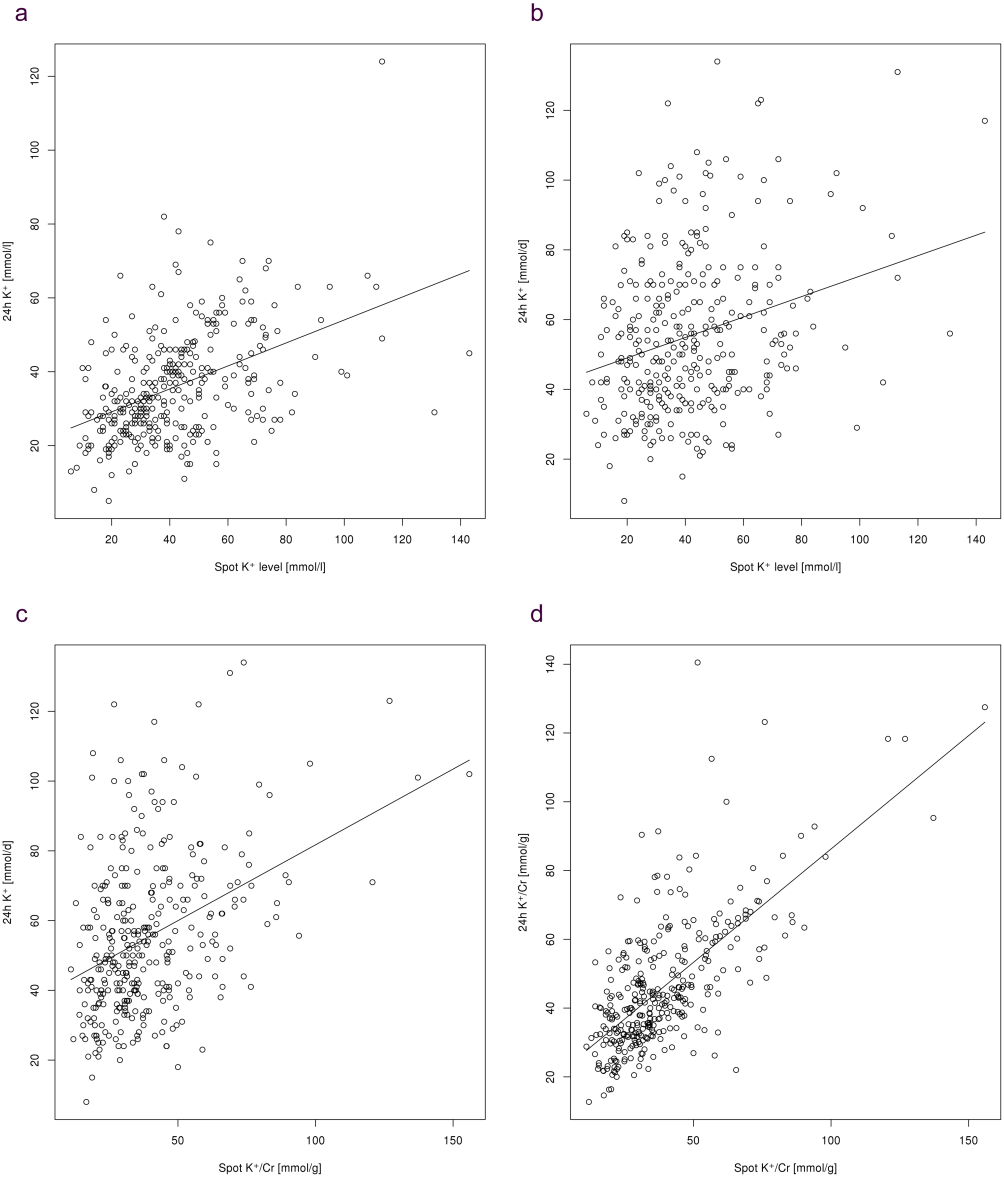
Correlations between spot urine and 24-hour urinary potassium measurements. 1a: Potassium level in the spot urine sample vs. potassium level in the 24-hour urine collection (r = 0.47, P<0.001). 1b: Potassium level in the spot urine sample vs. 24-hour urinary potassium excretion (r = 0.28, P<0.001). 1c: Potassium/creatinine ratio in the spot urine sample vs. 24-hour urinary potassium excretion (r = 0.38, P<0.001). 1d: Potassium/creatinine ratio in the spot urine sample vs. 24-hour urinary potassium/creatinine ratio (r = 0.69, P<0.001).

Five patients (1.3%) had missing laboratory data and were not included in the final analysis. When patients with oliguria/polyuria and inadequate urine collection were excluded, as per exclusion criteria in the study protocol (n = 43, or 11.2% of all patients), we found a significant correlation between potassium levels in spot urine samples and the 24-hour urine collection (r = 0.47; P<0.001) (Fig 1a). However, the potassium level in spot urine samples showed only a weak correlation with 24-hour urinary potassium excretion (r = 0.28, P<0.001) (Fig 1b). When the urinary potassium level was expressed as its ratio to the urinary creatinine level, the potassium/creatinine ratio in spot urine samples correlated with both 24-hour urinary potassium excretion (r = 0.38, P<0.001) (Fig 1c) and most strongly with 24-hour urinary potassium/creatinine ratio (r = 0.69, P<0.001) (Fig 1d).

As a secondary analysis, we also assessed whether patient exclusion due to too low/too much urine output or inadequate urine collection as per the study exclusion criteria affected these results. When all patients were included in the analysis, correlations were weaker but remained significant (Table 2). As some participants were treated with diuretics or might have had hyperglycemia, which might cause a sporadic increase in urinary potassium excretion and lead to a discrepancy between spot and 24-hour urinary potassium measurements, we also performed a secondary analysis after additionally excluding patients treated with loop diuretics or thiazides and those with diabetes, impaired fasting glucose or impaired glucose tolerance. Again, correlations were similar to those in the primary analysis (Table 2).

**Table 2:** Correlations between spot urine and 24-hour urine collection measurements.

Correlation	All patients (N = 377)	After exclusions as per study protocol (N = 334)	Additional exclusion of patients with diabetes/IFG/IGT and those on diuretics (N = 184)
**Potassium level in the spot urine vs. potassium level in the 24-hour urine collection**	r = 0.44, P<0.001	r = 0.47, P<0.001	r = 0.39, P<0.001
**Potassium level in the spot urine vs. 24-hour urinary potassium excretion**	r = 0.21, P<0.001	r = 0.28, P<0.001	r = 0.26, P<0.001
**Potassium/creatinine ratio in the spot urine vs. 24-hour urinary potassium excretion**	r = 0.33, P<0.001	r = 0.38, P<0.001	r = 0.36, P<0.001
**Potassium/creatinine ratio in the spot urine vs. 24-hr urinary potassium/creatinine ratio**	r = 0.66, P<0.001	r = 0.69, P<0.001	r = 0.64, P<0.001

IGF, impaired fasting glucose; IGT, impaired glucose tolerance.

### Estimation of 24-hour urinary potassium excretion based on potassium level in the spot urine sample

The mean measured 24-hour urinary potassium excretion (after patient exclusions as per the study exclusion criteria) was 55±22 mmol/d. The mean estimated 24-hour urinary potassium excretion in these patients was 47±24 mmol/d. The PAHO formula underestimated 24-hour urinary potassium excretion by mean 8.3±18 mmol/d, with 95% limits of agreement of -28 to +44 mmol/d. The correlation coefficient for the correlation between estimated and measured 24-hour urinary potassium excretion was r = 0.69 (P<0.0001).

**Fig 2:**
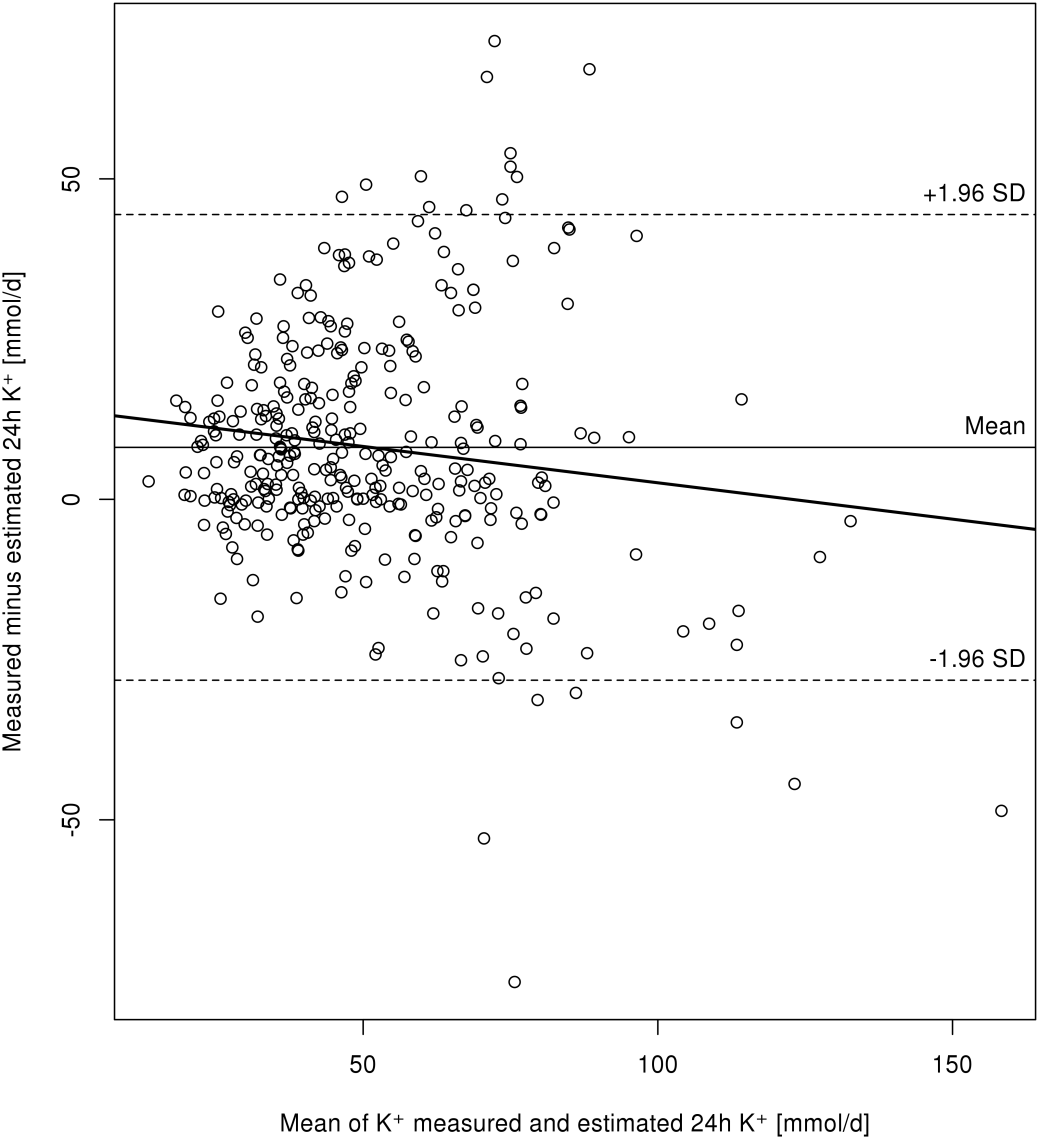
The Bland-Altman plot. The Bland-Altman plot showing the difference between measured and estimated (PAHO formula) 24-hour urinary potassium excretion plotted against the mean 24-hour urinary potassium excretion by the two methods (mmol/d). Dashed lines indicate 95% limits of agreement (±1.96 SD).

The regression line on the Bland-Altman plot (Fig 2) indicated that underestimation of 24-hour urinary potassium excretion by the PAHO formula decreased with higher actual 24-hour urinary potassium excretion values, although individual differences between the measured and estimated 24-hour urinary potassium excretion showed an increased scatter with 24-hour urinary potassium excretion of approximately ≥70 mmol/d. When patients treated with diuretics and those with a potential for hyperglycemia were excluded from the analysis, the mean bias was 8.0 mmol/d (95% limits of agreement -30 to 46 mmol/d).

### Diagnostic performance of the spot urine potassium/creatinine ratio and the estimated 24-hour urinary potassium excretion

**Fig 3:**
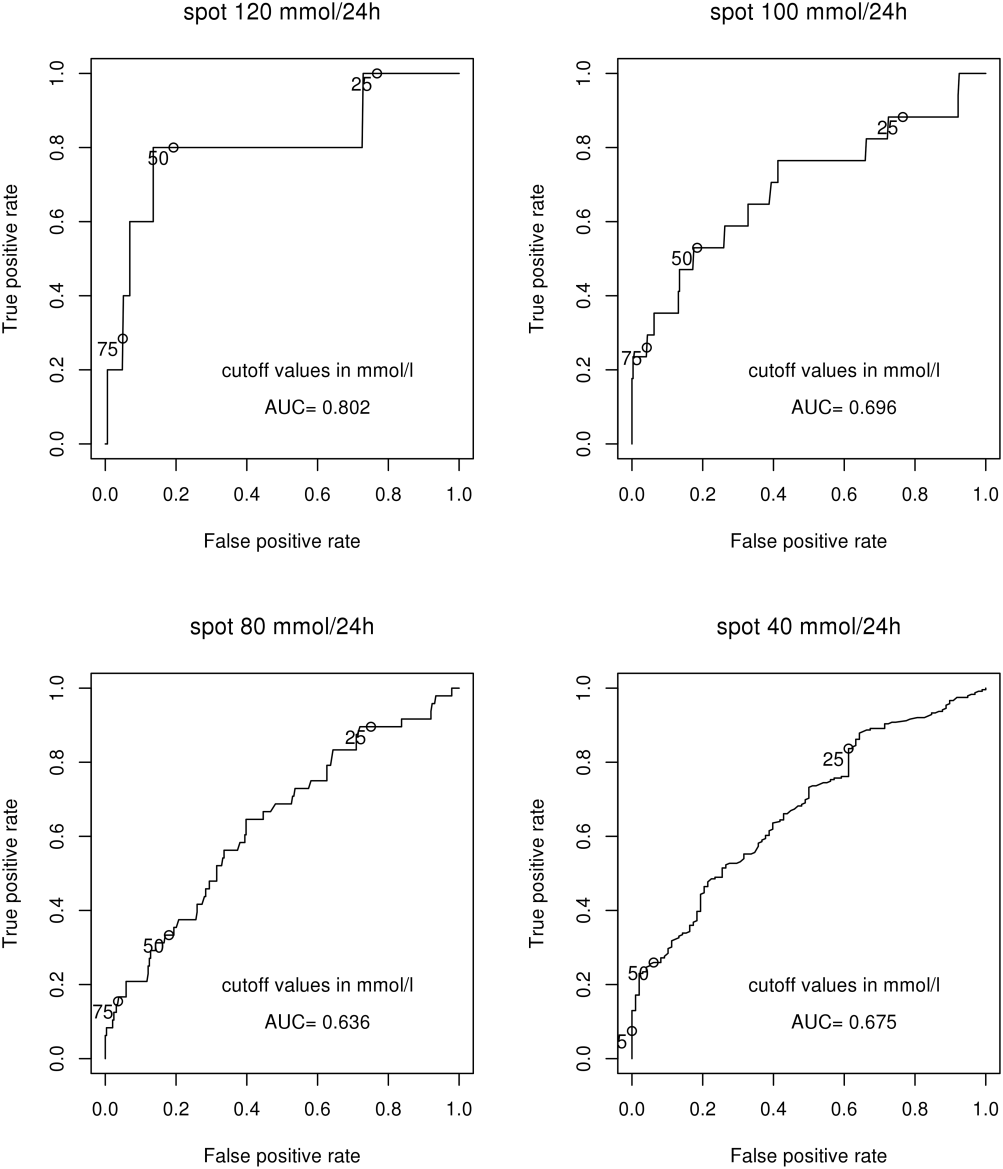
Receiver-operating characteristic (ROC) curves for the potassium/creatinine ratio in spot urine samples. The ROC curve for cut-off 24-hour urinary potassium excretion of 60 mmol/24 h was omitted for clarity. AUC, area under the curve.

Based on the ROC curves, the diagnostic performance of the spot urine potassium/creatinine ratio was borderline good only if 24-hour urinary potassium excretion was largely elevated (AUC 0.802 for the cut-off value of 120 mmol K^+^/24 h), but poor with lower values of 24-hour urinary potassium excretion (AUC 0.696 for 100 mmol K^+^/24 h, 0.636 for 80 mmol K^+^/24 h, 0.675 for 40 mmol K^+^/24 h) (Fig 3).

**Fig 4:**
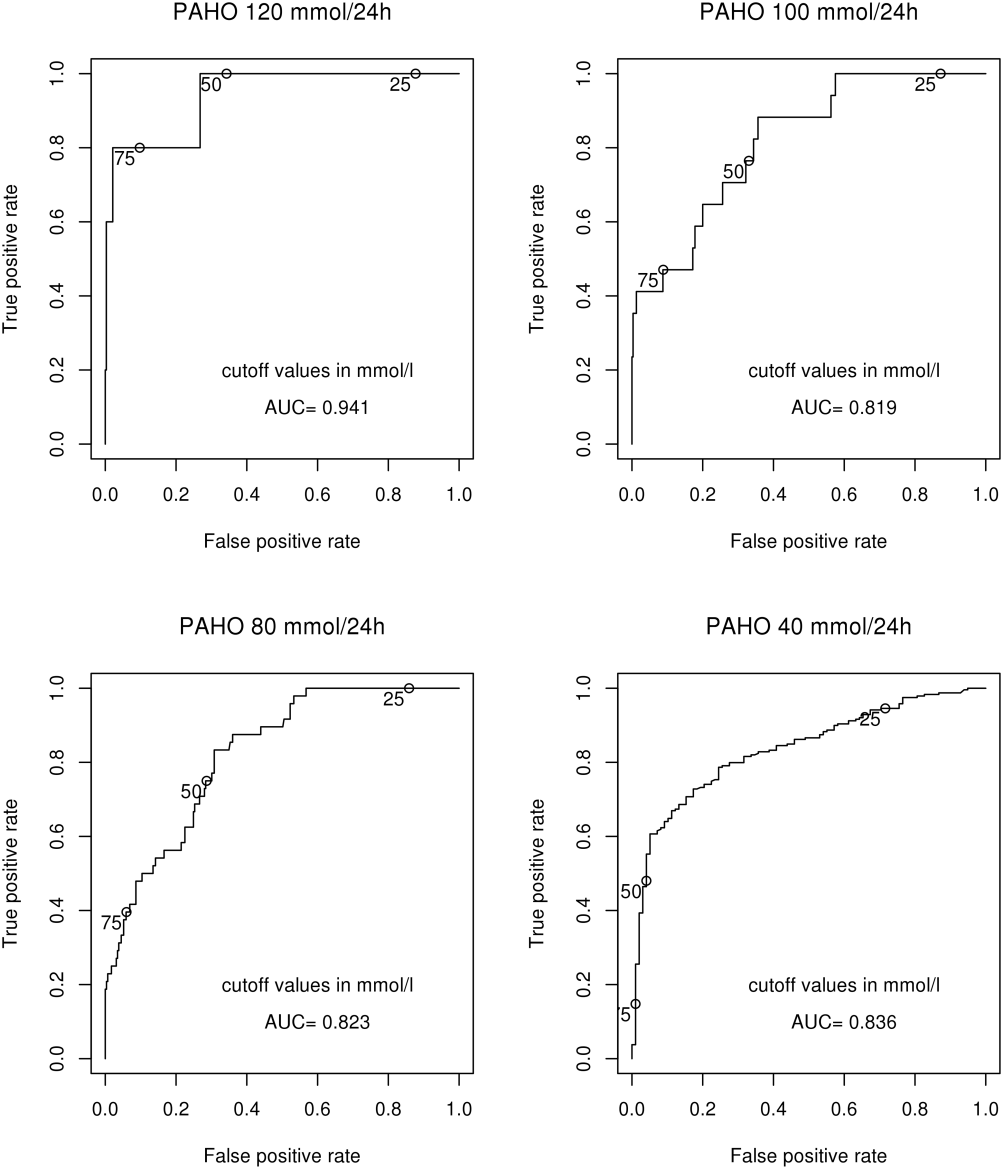
Receiver-operating characteristic (ROC) curves for the estimated 24-hour urinary potassium excretion. The ROC curve for cut-off 24-hour urinary potassium excretion of 60 mmol/24 h was omitted for clarity. AUC, area under the curve.

In contrast, the diagnostic performance of the estimated 24-hour urinary potassium excretion was excellent with values above 120 mmol/d and good with lower values (AUC 0.941 for the cut-off value of 120 mmol K^+^/24 h, 0.819 for 100 mmol K^+^/24 h, 0.823 for 80 mmol K^+^/24 h, 0.836 for 40 mmol K^+^/24 h) (Fig 4).

## Discussion

In contrast to previous studies, mostly performed in healthy young populations, we studied hypertensive patients referred to a tertiary care center. Our study was performed in real life clinical settings using standard clinical diagnostic approaches and laboratory methods, with no particular patient selection, and with routine inpatient oversight over the conditions of 24-hour urine and spot urine collection. The strength of our study is that in our analysis, we used not only the correlation coefficient but also the Bland-Altman approach, and the ROC curves to evaluate the diagnostic performance of spot urine potassium measurements.

We found that the spot urine potassium level alone was not useful as an index of 24-hour urinary potassium excretion, although it correlated with the potassium level in the 24-hour urine collection and the measured 24-hour urinary potassium excretion. However, the potassium to creatinine ratio in a spot urine sample correlated closely with the same ratio for 24-hour urine collection, thus representing a potentially useful measure of 24-hour urinary potassium excretion without the need to perform lengthy and cumbersome urine collections.

This adjustment for urinary creatinine level introduces a measure of the state of concentration or dilution of the urine, and it is used to estimate 24-hour urinary excretion of other analytes such as albumin [5]. Alternative ways of controlling for urinary potassium concentration were also reported in the literature (e.g., using potassium/urine specific gravity ratio), but found to be inferior to the potassium/creatinine ratio [14].

Our estimates of the correlation between the potassium/creatinine ratio in the spot urine and 24-hour urinary collection are in general agreement with the results of previous studies in the general population that showed good correlations between sodium/creatinine and potassium/creatinine ratios in spot urine and respective ratios in 24-hour urine [7,8]. However, most studies that compared spot urine and 24-hour urinary collections focused only on estimating 24-hour urinary sodium excretion as a measure of population salt intake [6,15].

Three formulas have been proposed to estimate 24-hour urinary potassium excretion based on potassium level measurement in a spot urine sample. Two of these formulas, developed for both sodium and potassium by Kawasaki et al. [7] and Tanaka et al. [8], were validated in healthy Asian populations. A simpler formula has been proposed by PAHO [9] and used in a number of previous studies [11,16,17]. Other formulas were also developed specifically for sodium but not potassium, such as the INTERSALT formula [18]. However, the Kawasaki and Tanaka formulas for sodium were shown to be inadequate in non-Asian populations [16,19]. Thus, for the purpose of the present analysis we chose the PAHO formula, which offers a simpler way to calculate the estimated 24-hour urinary potassium excretion.

### Timing of spot urine sampling

The optimal timing of spot urine sampling for the purpose of evaluating spot urine-based estimates of 24-hour excretion compared to the actual measured 24-hour urinary excretion has been debated in the literature [6,11,16]. In our study, spot urine sample was collected in the morning, at the time of initiation of 24-hour urine collection, similarly to some other studies [17] and also reflecting the routine diagnostic procedures in our hospital unit.

The timing of the spot urine sampling during the day has also varied between the published studies, an issue that may be important in the context of a circadian rhythm of urinary potassium excretion, which tends to be lower at night and higher during the day [8]. Some studies excluded the first morning urine to avoid measuring night-time potassium excretion [20]. Wang et al. [21] studied 4 timed-spot specimens and a 24-hour urinary collection obtained twice in a group of young adults. They found that urinary potassium excretion varied by timing of collection during 24 hours, with urinary potassium levels highest in the morning (55–64 mmol/l) and lowest overnight (30–36 mmol/l). When comparing two 24-hour urinary collections, the within person variance was 17%, and morning specimens were characterized by lower variance (25–26%) compared to evening specimens (31–40%). Thus, variability of urinary potassium excretion seems lower in the morning, while urinary potassium levels are higher at that time of the day, and this higher and more constant contribution of the morning urine to 24-hour urinary potassium excretion may possibly increase the chance of identifying increased potassium urinary excretion based on evaluation of morning spot urine samples.

### Limitations of 24-hour urine collection

Although 24-hour urinary collection is considered the gold standard for assessing urinary sodium and potassium excretion, this approach has significant limitations [22] and may be inadequately reliable at the individual level [23]. The need to collect complete urine output over a 24-hour period poses a high burden on patients. This results in low participation rates noted in some population studies, and potential inaccuracy of collection completeness, with both under- and overcollection reported [4,6]. Although the participation rate was not an issue in the inpatient clinical setting of our study, the 24-hour urinary collection could still be inaccurate. The biochemical method of administering para-aminobenzoic acid (PABA) orally and measuring urinary PABA level to evaluate urine collection completeness [4] was not feasible in our study for logistic reasons and due to additional costs it would incur. Instead, we used a less precise but more feasible alternative approach of evaluating 24-hour urine volume and urinary creatinine excretion, which is reasonably constant for a given individual and depends mainly on lean body mass, age, and gender [4].

### The Bland-Altman approach

The Bland-Altman approach is considered a better method to assess the agreement between two methods of measurement than correlation, particularly as related to individual patient management [11,16,24]. With this approach, the measurement differences between the two methods are plotted against the mean of the two measurements to visualize how much the measurement by the new method (i.e., estimation using spot urine) differs from the one by the reference method (i.e., measurement of the actual 24-hour urinary excretion). If the 95% limit of agreement, i.e. maximum difference between the measurements by the two methods in 95% of cases, is considered clinically acceptable, the two methods may be used interchangeably.

In our study, the Bland-Altman approach showed that the PAHO formula slightly underestimated 24-hour urinary potassium excretion. The mean difference between the measured and estimated values in our study population was 8.3 mmol per day. In individual patients, this error of estimation may be expected to lie somewhere between underestimation by 44 mmol per day and overestimation by 28 mmol per day in 95% of cases.

An estimate of potassium excretion calculated from a spot urine sample cannot correlate precisely with the actual 24-hour excretion since spot urine reflects potassium excretion over a shorter time period of only a few hours, and urinary potassium excretion changes in relation to many factors and is not constant throughout 24 hours. One such factor discussed previously is a circadian rhythm of potassium excretion, with lower urinary potassium levels noted during the night [21]. Potassium excretion also depends on its intake, sodium excretion, effect of aldosterone, and urine flow [25]. Thus, wide 95% limits of agreement by the Bland-Altman method are to be expected. In the study by Doenyas-Barak et al. [26], the evening sample correlated with the 24-hour urinary potassium excretion better than the morning sample (r = 0.75 vs. r = 0.53). Based on this fact, these authors suggested that diurnal aldosterone fluctuations are most important in regard to potassium excretion. It can also be speculated that in patients with PHA, autonomic excess aldosterone secretion will be an even more important and perhaps also a more constant factor, reducing the variability of potassium excretion over time.

Our results may be compared with other studies that used the Bland-Altman approach for potassium measurements in spot urine samples in comparison to actual 24-hour urinary potassium excretion in healthy subjects. In the study by Doenyas-Barak et al. [26] using a modification of the PAHO formula, the mean bias estimated using the Bland-Altman method was +11 mmol/d, with 95% limits of agreement from -33 mmol/d to +55 mmol/d. In the study by Mizéhoun-Adissoda et al. [17], the mean bias for the PAHO formula was -13.4 mmol/d, with 95% limits of agreement from -53 to +23 mmol/d. The authors of these studies concluded that this approach could be used to estimate 24-hour urinary potassium excretion. In contrast, Hooft van Huysduynen et al. [20] in a study in young healthy Danish women concluded that it was not possible to accurately predict 24-hour urinary potassium excretion based on spot urine measurements using the Tanaka formula.

Only few studies were performed specifically in hypertensive patients, mostly focusing on the estimation of sodium excretion based on spot urine measurements [27–29]. To the best of our knowledge, no previous study evaluated the Bland-Altman approach for estimating 24-hour urinary potassium excretion based on spot urine measurements in hypertensives.

### Analysis of the receiver-operating characteristic curves

To further evaluate the diagnostic performance of the spot urine potassium/creatinine ratio and the estimated 24-hour urinary potassium excretion, we calculated AUC values for respective ROC curves at various cut-off levels of the actual 24-hour urinary potassium excretion. Although the potassium/creatinine ratio in a spot urine sample correlated well with 24-hour urine potassium/creatinine, it had poor discriminative value based on the ROC curve analysis unless 24-hour urinary potassium excretion was largely elevated. Based on the AUC values obtained, the diagnostic performance of spot urine potassium/creatinine ratio was good only if 24-hour urinary potassium excretion exceeded 120 mmol/day. In contrast, the diagnostic performance of the 24-hour urinary potassium excretion estimated by the PAHO formula was excellent with 24-hour urinary potassium excretion at 120 mmol/d and good with lower values down to 40 mmol/d.

These findings might be of potential importance when considering use of spot urine measurements to identify inappropriate renal potassium loss in clinical practice. Inappropriate hyperkaliuria has been defined as an increased urinary potassium concentration in the presence of hypokalemia, if unprovoked by diuretic use [30]. Threshold values were given in the literature for 24-hour urinary collection, and also for urinary level measurements (e.g., in spot urine) or as a spot urine potassium-to-creatinine ratio, but not for the estimated 24-hour urinary excretion. When referring specifically to spot urine measurement, a threshold urinary potassium level of 30 or 40 mmol/L has been indicated as suggesting renal potassium loss [31]. When expressed as the urine potassium-to-creatinine ratio from a spot urine specimen, a ratio greater than 1.5 mmol/mmol (13 mmol/g) was suggested as indicative of renal potassium wasting [32].

Based on 24-hour urinary collection, inappropriate renal potassium loss has been conventionally defined as excretion of more than 30 mmol of potassium per day. Similar criteria could be probably used for estimated potassium excretion if this estimation were shown to be precise enough to have an adequate diagnostic value. We demonstrated that the diagnostic value of 24-hour urinary potassium excretion estimated using the PAHO formula was excellent for largely elevated urinary potassium excretion and remained good with lower values down to 40 mmol/d.

In addition, our ROC curve analysis findings indicate that for the purpose of identifying hyperkaliuria, the approach based on estimating 24-hour urinary potassium excretion using the PAHO formula might be more useful that an approach based on determination of the potassium/creatinine ratio in a spot urine sample and using a specific threshold value, such as one suggested in the literature.

### Study limitations

The study population included too few patients with PHA to allow meaningful evaluation of the latter group or to compare patients with essential hypertension and PHA. We did not control for such factors as potassium intake from diet and the daily level of exercise which may affect urinary potassium excretion, although it may be assumed that in the hospital setting, variability of these factors is reduced compared to outpatient conditions.

We cannot rule out over- or under-collection of 24-hour urine but this is a limitation of 24-hour urinary assessment method and of the reasons why an alternative approach based on spot urine measurements is being investigated. To reduce error related to inaccuracy of 24-hour urine collection completeness, we excluded patients with 24-hour urine output or creatinine excretion beyond the defined limits.

We relied on a single 24-hour urine collection and single morning spot urine in each patient, and we did not evaluate repeated measurements of the spot urine potassium to creatinine ratio. It has been postulated that repeated measurements of either spot or 24-hour urine over several days may allow more reliable assessment of sodium/potassium excretion/intake at the individual level. However, increasing the number of spot urine collections will increase the complexity of the overall diagnostic approach. Doenyas-Barak et al. [26] evaluated four scheduled spot urine samples throughout the day to estimate 24-hour urinary sodium and potassium excretion. For potassium, correlation for the 4-spot estimate (r = 0.76) was not better than for the best single spot (at 16:00, r = 0.75), although better than for the morning urine (r = 0.53). However, estimates using the Bland-Altman method were provided only for 4-spot estimate, so it is difficult to assess whether using four urine samples compared to just one had any incremental value in terms of predicting individual 24-hour urinary potassium excretion.

The PAHO formula requires measurement or estimation of 24-hour creatinine excretion. In this study, we used actual measured 24-hour creatinine excretion, which likely reduced the error of estimating 24-hour urinary potassium excretion. Similar approach was used in other studies [11,16]. However, since the purpose of the spot urine test is to eliminate the need for a 24-hour urine collection, a reasonable estimation of 24-hour urinary creatinine excretion is necessary, and the latter has been noted to vary by age, sex, body weight, ethnicity, and other factors. Currently available prediction equations for 24-hour urinary creatinine may have some limitations, and thus separate research is needed to develop the best approach to estimate 24-hour urinary creatinine excretion based on readily available clinical parameters [19,33].

## Conclusions

We found that although the potassium/creatinine ratio in a spot urine sample was not a very precise measure of individual 24-hour urinary potassium excretion in hospitalized hypertensive patients, it might serve as a marker of a significantly increased urinary potassium excretion that might be potentially useful for the purpose of rapid identification of hyperkaliuria induced by PHA without the need for performing cumbersome 24-hour urine collection. We also found that a simple formula allows estimation of the 24-hour urinary potassium excretion based on spot urine measurements with reasonable clinical accuracy, particularly for the purpose of identification of an increased urinary potassium loss. In summary, despite the established limitations of spot urine as a measure of overall 24-hour urine output and known diurnal variation of urinary potassium excretion, evaluation of the potassium level in routine spot urine samples may be considered a potentially useful screening test when investigating hypertensive patients.

## Supporting information

S1 Dataset Dataset. Database with raw data (as an Excel spreadsheet).
(XLSX)
